# Predicting mortality and re-hospitalization for heart failure: a machine-learning and cluster analysis on frailty and comorbidity

**DOI:** 10.1007/s40520-023-02566-w

**Published:** 2023-10-18

**Authors:** Chukwuma Okoye, Tessa Mazzarone, Filippo Niccolai, Leonardo Bencivenga, Giulia Pescatore, Maria Giovanna Bianco, Cinzia Guerrini, Andrea Giusti, Daniela Guarino, Agostino Virdis

**Affiliations:** 1 Geriatrics Unit, Department of Clinical and Experimental Medicine, University of Pisa, Via Paradisa, 2, 56124 Pisa, Italy; 2 Department of Neurobiology, Care Sciences and Society, Department of Geriatrics Aging Research Center, Karolinska Institutet, Stockholm University, Stockholm, Sweden; 3 School of Medicine and Surgery, University of Milano-Bicocca, Milan, Italy; 4 Department of Translational Medical Sciences, University of Naples Federico II, Naples, Italy

**Keywords:** Heart failure, Older adults, Clustering, Frailty, Comorbidities

## Abstract

**Background:**

Machine-learning techniques have been recently utilized to predict the probability of unfavorable outcomes among elderly patients suffering from heart failure (HF); yet none has integrated an assessment for frailty and comorbidity. This research seeks to determine which machine-learning-based phenogroups that incorporate frailty and comorbidity are most strongly correlated with death or readmission at hospital for HF within six months following discharge from hospital.

**Methods:**

In this single-center, prospective study of a tertiary care center, we included all patients aged 65 and older discharged for acute decompensated heart failure. Random forest analysis and a Cox multivariable regression were performed to determine the predictors of the composite endpoint. By k-means and hierarchical clustering, those predictors were utilized to phenomapping the cohort in four different clusters.

**Results:**

A total of 571 patients were included in the study. Cluster analysis identified four different clusters according to frailty, burden of comorbidities and BNP. As compared with Cluster 4, we found an increased 6-month risk of poor outcomes patients in Cluster 1 (very frail and comorbid; HR 3.53 [95% CI 2.30–5.39]), Cluster 2 (pre-frail with low levels of BNP; HR 2.59 [95% CI 1.66–4.07], and in Cluster 3 (pre-frail and comorbid with high levels of BNP; HR 3.75 [95% CI 2.25–6.27])).

**Conclusions:**

In older patients discharged for ADHF, the cluster analysis identified four distinct phenotypes according to frailty degree, comorbidity, and BNP levels. Further studies are warranted to validate these phenogroups and to guide an appropriate selection of personalized, model of care.

**Supplementary Information:**

The online version contains supplementary material available at 10.1007/s40520-023-02566-w.

## Introduction

The aging of the global population is accompanied by an increasing prevalence of individuals with multiple comorbidities, burdening the worldwide healthcare systems’ sustainability. Heart failure (HF) is a chronic disabling condition, whose prevalence exponentially increases in late life, and represents a leading cause of morbidity and mortality [1]. It has been estimated that 4 out of 5 individuals with HF are 65 years or older [2]; however, scientific evidence indicates that advanced chronological age alone is not necessarily a risk factor for adverse events. Rather, other factors such as functional ability, inflammatory status, lifestyle and other interconnected factors strongly contribute to the higher heterogeneity observed among older adults, and influence their prognosis [3–5]. Tailoring treatment to the individual patient's needs is, thus, of paramount importance, both to offer them the most appropriate care and to also avoid incurring high healthcare costs unnecessarily. In this regard, older patients with HF are more prone to experience a series of adverse clinical outcomes, including re-hospitalizations for acute decompensated HF (ADHF) and death [3]. Current guidelines [2] recommend ascertaining the presence of precipitating factors in older patients with acute heart failure since they substantially influence the 90-day mortality of ADHF patients [6]. In the past years, several methodological approaches and models have been proposed to predict post-discharge risk in HF patients by using a combination of demographic, clinical, and easily obtainable data, to achieve the highest predictive power [4, 7–10]. However, most of the proposed models seem to underestimate the clinical impact of frailty on mortality in older patients with HF [11]. Indeed, frailty is a common condition in older patients with HF [5] and is associated with worse outcomes [12–15]. Although uncertainty persists as to which tool is the most appropriate for assessing frailty in this population and how multidimensional geriatric parameters correlate with prognosis in older patients with HF [16–18], the importance of frailty assessment for ADHF patients has become indisputable. As matter of the fact, a position paper from the American Heart Association (AHA), American College of Cardiology, and American Geriatrics Society states that future guidelines should consider the assessment of frailty domains as a reliable indicator of a patient's biological age and health status [19]. Recently, the use of statistical learning algorithms applied to dense phenotypic data have been proposed to improve classification of heterogeneous clinical syndromes, with the objective of a patient-centered therapeutic approach. Thus, machine-learning techniques have been also employed to predict the risk of developing adverse outcomes in patients with HF [20–23]; however, none of these included a frailty assessment. The present study aimed to identify the independent risk factors for 6-month re-hospitalization for ADHF or death after discharge, and to determine the clinical phenotype of older patients at greater risk of developing the composite endpoint using an unsupervised machine-learning technique.

## Methods

Patients aged 65 or older discharged from a geriatric unit of a tertiary care hospital with diagnosis of acute decompensated heart failure (ADHF) from January 1st, 2018, and September 30th, 2019, were retrospectively included, without any exclusion criteria. At hospital admission, all the patients had undergone a comprehensive geriatric assessment (CGA) [24] including: cognitive evaluation using the Short Portable Mental Status Questionnaire (SPMSQ) [25], evaluation of basic (ADL) [26], and instrumental (IADL) [27] activities of daily living. Comorbidities burden was evaluated through the Charlson Comorbidity Index [28]. The frailty degree was evaluated through the Clinical Frailty Scale (CFS) [29]. The Clinical Frailty Scale is a judgment-based visuo-analogic frailty tool that evaluates specific domains including comorbidity, function, and cognition to generate a frailty score ranging from 1 (very fit) to 9 (terminally ill). All the patients had also undergone complete blood tests: creatinine and brain natriuretic peptide (BNP) were routinely evaluated in all patients at the time of discharge. We defined the primary outcome as a composite of re-hospitalization for management of HF, or all-cause death within six months following discharge. Mortality rate and HF re-hospitalization was assessed by phone call and computerized hospital archive. Time-to-event was measured as the number of months from hospital discharge to the date of first event occurrence. Study participants were right censored at the time of their last follow-up for clinical outcomes or at 6 months. Follow-up was recorded in all patients. The study complied with the Declaration of Helsinki and was approved by the local Ethics Committee (Tuscany Regional Ethics Committee for the Clinical Experimentation: FUN-sc 23956).

### Statistical analysis

Continuous variables were presented as mean and standard deviation, ordinal variables as median and interquartile range (IQR), and categorical variables as number of observations and percentage. Mann–Whitney and chi-square tests were used for multiple comparisons.

### Cox Regression analysis

Univariate and multivariate Cox regression analyses were performed to identify clinical and biochemical factors associated with the pre-specified endpoint (6-months mortality or re-hospitalization for HF). Univariate Cox regression was performed with the following continuous and categorical covariates: age, sex, hypertension, type 2 diabetes mellitus, atrial fibrillation, chronic obstructive pulmonary disease, creatinine, ejection fraction, history of stroke, anemia, coronary artery disease (CAD), Clinical Frailty Scale (CFS), Charlson Comorbidity Index (CCI), BNP. The multivariable Cox regression was then performed among statistically significant covariates of the univariate analysis. A receiver operating curve (ROC) was performed to determine the AUC of the composite endpoint for the clinically relevant and significant determinant of the aforementioned Cox regression.

### Random forest analysis

To explore the predictive capacity of the machine-learning approach, a random forest analysis for feature selection was conducted. The dataset was divided into distinct training and testing sets to evaluate the model's generalization performance. The training set comprised 70% of the data, while the remaining 30% constituted the testing set. The target variable, “six-month endpoint” was separated from the predictor variables to ensure that the model's predictions were unbiased and reliable. Two separate random forest models with five-hundred trees were trained: one utilizing the training dataset (“training model”) and the other using the testing dataset (“testing model”). The models were trained to predict the composite endpoint of 6-month mortality or re-hospitalization for heart failure. For each model, the hyperparameters were tuned to optimize the model's performance. The number of decision trees in the ensemble, known as the “n_trees” parameter, was determined using a grid search and cross-validation procedure. This parameter was selected to strike a balance between predictive accuracy and computational efficiency. During the training process, the out-of-bag (OOB) estimate of the error rate was calculated.

### Determination of variable importance

The assessment of variable importance was a pivotal aspect of our analytical approach, aimed at elucidating the factors that significantly contribute to the predictive performance of the Random Forest model. We employed the ‘importance()’ function, inherent to the Random Forest methodology, to calculate importance scores for each predictor variable. These scores reflect the magnitude of each variable's influence on the model's predictions. Higher importance scores indicate variables that exert a more substantial impact on the predictive accuracy of the model. The variables were ranked based on their respective importance scores, providing a hierarchy of their contribution to the predictive task. Variables with higher importance scores assumed greater prominence in the model's decision-making process.

### K-means clustering analysis

K-means is a centroid-based clustering algorithm that performs by partitioning a dataset into k clusters by minimizing the sum of squared distance in each cluster. Both in k-means and hierarchical clustering, the number of clusters was chosen by using the analytical “silhouette approach”, by deriving the average silhouette width for a number from 1 to 10 clusters. A high average silhouette width indicates a good clustering. The optimal number of clusters k is the one that maximizes the average silhouette over a range of possible values for k. Having specified the number of clusters k, each patient was assigned to the nearest centroid, and the cluster centroid was updated sequentially. This process was repeated until the sum of squared distance was minimized and each patient was assigned to one cluster based on Euclidean distance. A visual presentation of the clustering was presented.

### Hierarchical agglomerative clustering

To further strengthen the findings from the k-means clustering, we conducted a hierarchical agglomerative clustering, performed on the same predictors as the first analysis, using an agglomerative nesting algorithm. At each iteration, the two most similar points merge into a single branch of a dendrogram, resulting in branch formations of increasingly larger clusters. Ultimately, all points are merged into a single branch, which can be cut at a specified distance to form clusters. The distance at which the dendrogram is cut can be determined by using various approaches, including visually assessing the natural distribution of the data, optimizing cluster-wise distance metrics or reflecting underlying biological properties. One of the key advantages of using dendrograms is the absence of pre-specified cluster numbers, facilitating interpretability and visual analysis. Compared to other clustering methods, dendrograms are simple to conceptualize and easy to interpret visually. Ward’s method was used as a linkage criterion. The result of clustering was represented using a dendrogram.

### Relationship between clusters and the 6-month composite endpoint

We utilized the phenogroups derived from k-means clustering to conduct comparative analyses across outcome measures, sociodemographic factors, and clinical variables within each cluster. The risk of mortality was evaluated using a Kaplan–Meier estimator. After checking the proportional hazards assumption using Schöenfeld residuals, the hazard ratio (HR) and 95% confidence interval (95% CI) of mortality was calculated for the clinical endpoint and each cluster, using the cluster with the lowest risk as a reference. Univariable and multivariable models were performed, the latter being age- and sex adjusted. All statistics were performed using R version 4.0.2 (The R Foundation for Statistical Computing, Vienna, Austria, 2020) using the packages “cluster”, “factoextra”, “survival” and “surviminer” “ggplot2”, “dplyr”,”randomForest”.

## Results

We identified 571 patients hospitalized with acutely decompensated heart failure over the study period, of whom 313 (54.8%) were female. The mean age was 86.3 years (SD 6.2). Overall, patients presented a high burden of comorbidities [median Charlson Comorbidity Index 6 (IQR 2)], and a moderate-to-high degree of frailty (median CFS 6 [IQR 4]).

### Cox regression analysis and predictors’ area under the curve

As shown in Table 1, by stepwise multivariate Cox regression analysis, BNP (HR 1.00 [95% CI 1.00–1.01]), CFS (HR 1.40 [95% CI 1.27–1.54]), and CCI (HR 1.12 [95% CI 1.02–1.22]) emerged as determinants of 6-month mortality risk or re-hospitalization for HF. As secondary analysis, CFS resulted to exert a stronger capacity to predict the composite endpoint, compared to CCI and BNP level (respectively, AUC 0.702 [95% CI 0.659–0.745], 0.581 [95% CI 0.534–0.628] and 0.597 [95% CI 0.550–0.644]).

**Table 1:** Determinants or HF re-hospitalization or death. Stepwise Cox Regression Analysis

	Beta	S.E	H.R	95% CI		*p* value
				Lower	Upper	
**Univariate**						
Sex	− 0.23	0.21	0.78	0.52	1.19	0.26
Age	0.04	0.01	1.05	1.01	1.08	0.003
Hypertension	− 0.30	0.22	0.74	0.48	1.14	0.17
Atrial fibrillation	− 0.28	0.20	0.75	0.50	1.12	0.16
T2DM	− 0.21	0.20	0.80	0.54	1.20	0.80
COPD	0.17	0.22	1.19	0.77	1.84	0.42
CAD	− 0.23	0.20	0.78	0.52	1.18	0.25
Stroke	0.10	0.29	1.11	0.62	1.98	0.71
Anemia	0.07	0.21	1.07	0.70	1.64	0.72
Creatinine	0.11	0.06	1.11	0.99	1.25	0.06
Ejection fraction	− 0.18	0.01	0.98	0.97	0.98	0.005
BNP	0.00	0.00	1.00	1.00	1.00	< 0.001
CFS	0.32	0.05	1.38	1.25	1.53	< 0.001
CCI	0.10	0.04	1.11	1.01	1.21	0.018
**Multivariable (Step 7)**						
Age	0.04	0.01	1.04	1.01	1.08	0.005
CCI	0.11	0.04	1.12	1.02	1.22	0.009
CFS	0.33	0.05	1.45	1.31	1.61	< 0.001
Ejection fraction	− 0.01	0.01	0.99	0.97	1.01	0.26
BNP	0.00	0.00	1.00	1.00	1.00	< 0.001

*T2DM*: Type 2 Diabetes Mellitus; *COPD*: Chronic Obstructive Pulmonary Disease;* CAD*: Coronary Artery Disease; *BNP*: Brain Natriuretic Peptide; *CCI*: Charlson Comorbidity Score; *CFS*: Clinical Frailty Scale

### Random forest analysis

**Fig. 1:**
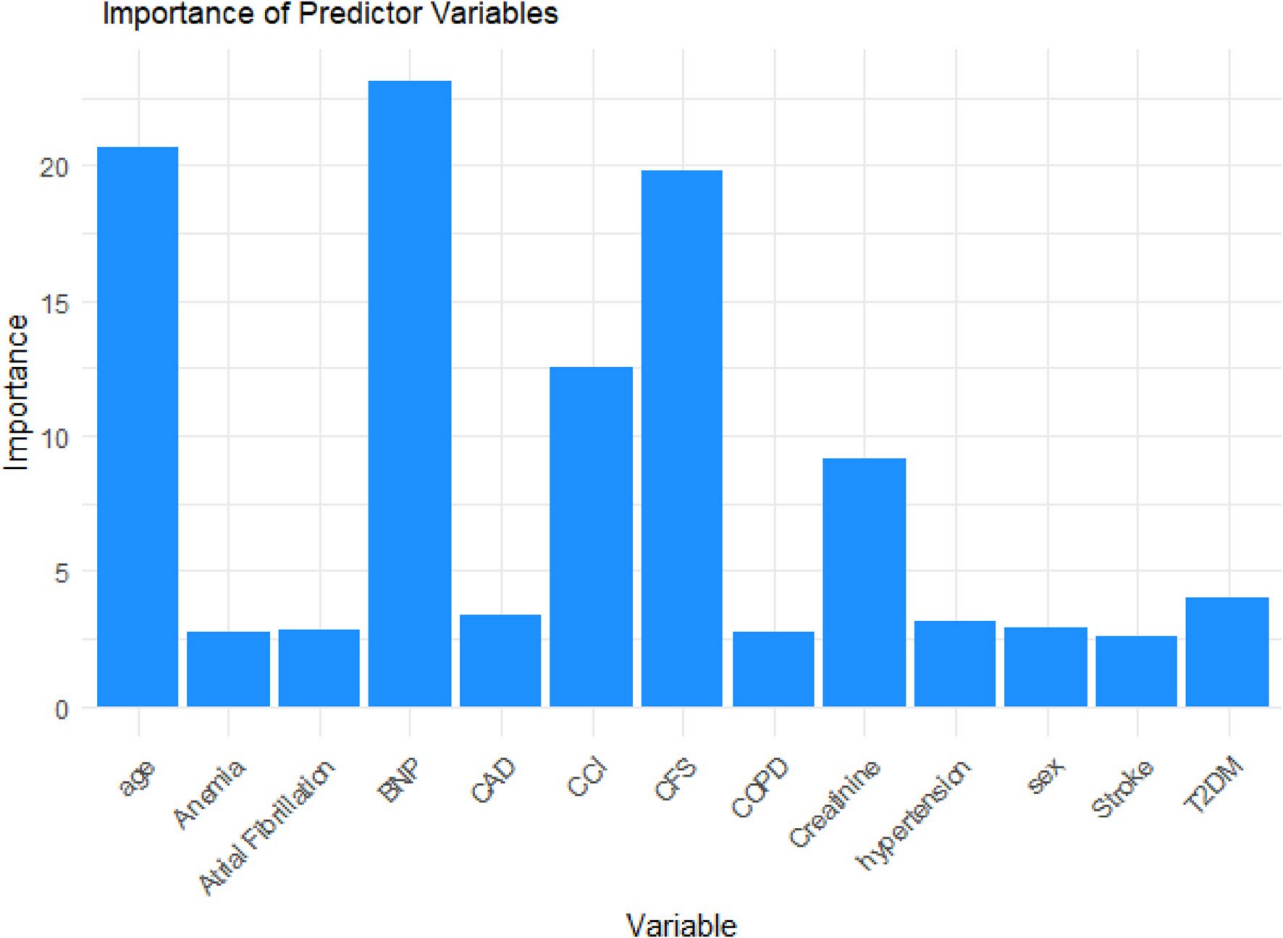
Random forest analysis: importance values bar plot

By the random forest model, we found the highest importance values for BNP with an importance value (IV) of 23.10, signifying its substantial impact on the predictive accuracy of the model; Age with an of 20.65, CFS 19.82 and CCI 12.53. Creatinine levels had an importance value of 9.13, reflecting their substantial contribution to the model's predictive capacity (Fig. 1). Other variables, including hypertension, diabetes, atrial fibrillation (AF), chronic obstructive pulmonary disease (COPD), coronary artery disease (CAD), stroke, and anemia, exhibit relatively lower importance values. In our analysis, the OOB estimate of error rate was found to be 2.26%. This indicates that, on average, the model correctly classified approximately 97.74% of the data points in the training dataset.

### K-means clustering analysis

**Fig. 2:**
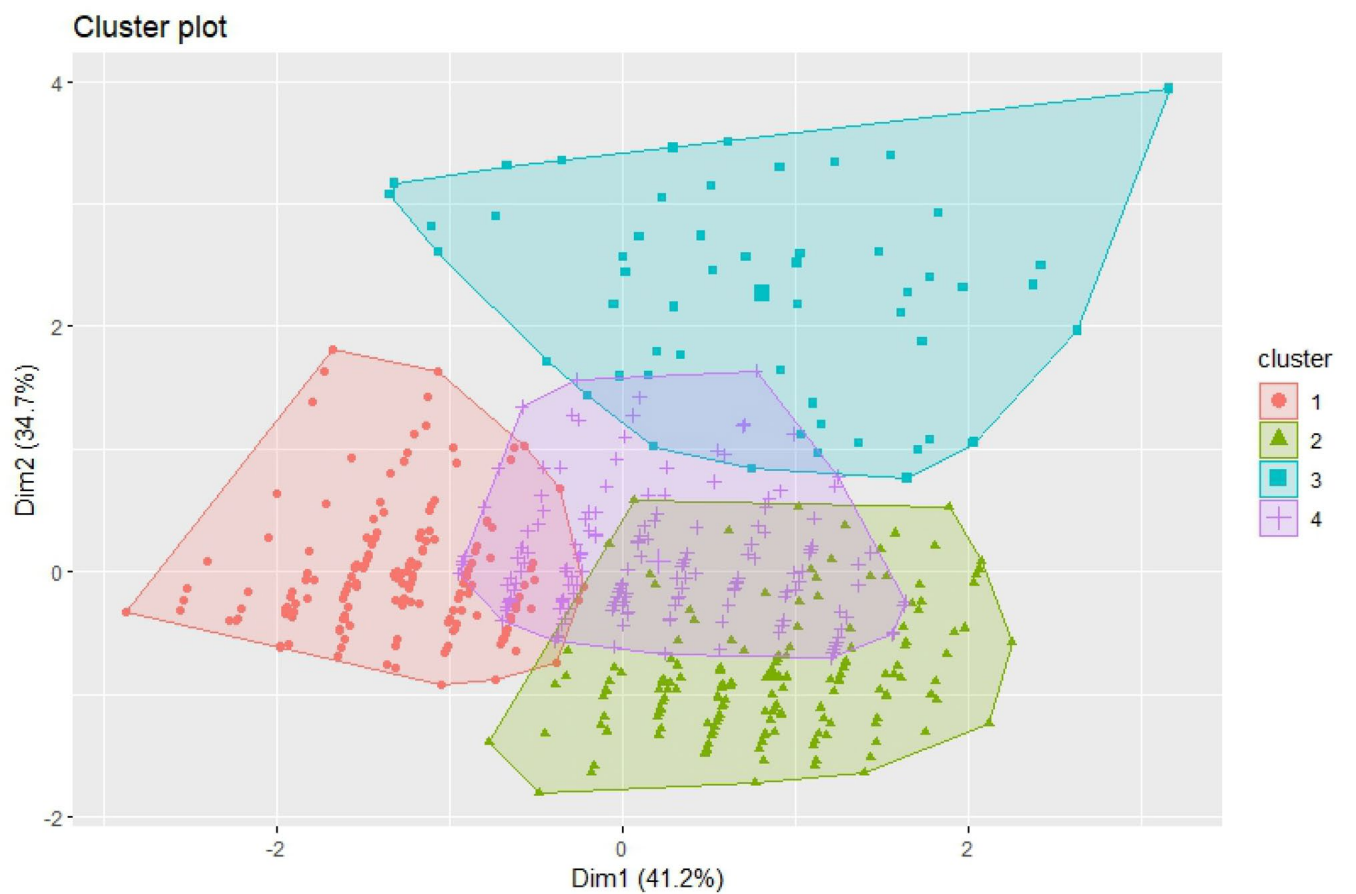
Cluster plot of the frailty, BNP and comorbidity-based phenogroups

The k-means clustering algorithm was performed to verify the possible segregation of ADHF patients in different clusters (Fig. 2). Based on BNP level, CFS, and CCI, the algorithm identified four different phenogroups (see Table 2). Cluster 1 was composed of very frail patients; Cluster 2 included pre-frail-to frail patients with an intermediate BNP; Cluster 3 comprised pre-frail-to-frail patients with high levels of BNP; and Cluster 4 was composed of non-frail patients.

**Table 2:** Comparison between different clusters

	All patients *N* = 571	Cluster 1 *N* = 174	Cluster 2 *N* = 169	Cluster 3 *N* = 59	Cluster 4 *N* = 169	*p* value
Female (%)	313 (54.8)	104 (59.7)	115 (68.0)	27 (45.7)	67 (39.6)	< 0.001
Age mean, years (SD)	86.3 (6.3)	88.0(5.7)	85.5(7.1)	85.5(7.1)	83.9(5.9)	< 0.001
Median LOS	6 (3)	6 (3)	6 (4)	6 (4)	6 (3)	0.43
Hypertension (%)	396 (69.3)	121(69.5)	104 (61.5)	40 (67.8)	131(77.5)	0.009
Atrial fibrillation (%)	347 (60.7)	109 (69.6)	102 (60.3)	39 (66.1)	97 (57.4)	0.61
Type 2 diabetes mellitus	176 (30.8)	62 (35.6)	47 (27.8)	17 (28.8)	50 (29.6)	0.35
COPD (%)	163 (28.5)	45 (25.8)	41 (24.2)	19 (32.2)	58 (34.7)	0.14
Stroke (%)	78 (13.8)	32 (20.1)	20 (11.8)	10 (16.9)	13 (7.8)	0.006
Dementia (%)	175 (30.6)	79 (45.4)	67 (39.6)	17 (28.8)	12 (7)	< 0.001
Kidney chronic disease	235 (41.5)	60 (34.4)	70 (41.4)	42 (71.2)	63 (37.7)	< 0.001
Coronary artery disease	211 (37.3)	49 (28.1)	70 (41.2)	30 (50.8)	62 (37.7)	0.010
Malnourishment or at risk	174 (30.4)	62 (35.6)	65 (38.4)	27 (45.7)	20 (12.2)	< 0.001
Anemia (%)	172 (30.3)	49 (28.6)	52 (30.7)	26 (44.0)	45 (26.9)	0.09
ADL median (IQR)	3 (5)	2 (3)	2 (3)	3 (5.5)	6 (3)	< 0.001
IADL median (IQR)	1 (4)	0 (1)	1 (2)	1 (3)	5 (4)	< 0.001
CFS median (IQR)	6 (4)	7 (2)	6 (1)	6 (4)	2 (1)	< 0.001
CCI median (IQR)	5 (4)	7 (3)	3 (2)	4 (3)	5 (3)	< 0.001
Creatinine median (IQR)	1.22 (0.85)	1.19 (0.84)	1.03 (0.79)	1.59 (0.8)	1.22 (0.69)	< 0.001
Ejection fraction (%)	50 (24)	59 (24)	55 (21)	41 (25)	55 (22.5)	< 0.001
HFpEF	208 (51.4)	58 (50.8)	64 (57.1)	14 (28.5)	72 (56.6)	
HFmrEF	103 (25.5)	26 (25)	23 (13.6)	16 (27.1)	35 (27.5)	
HFrEF	92 (22.7)	28 (16.1)	25 (14.8)	19 (32.2)	20(15.7)	0.021
BNP median, pg/ml (IQR)	650 (931)	592 (723)	593 (743)	4100 (1585)	516 (698)	< 0.001
Mortality (%)	215 (37.7)	91 (52.3%)	65 (38.5)	29 (49.2)	30 (17.8)	< 0.001

Continuous variables are expressed as mean SD or median with IQR properly

*ADL* activities of daily living, *IADL* instrumental activities of daily living, *SPMSQ* Short Portable Mental Status Questionnaire, CIRS-C Cumulative Illness Rating Scale, *CFS* Clinical Frailty Scale, *BNP* Brain natriuretic peptide

**Fig. 3:**
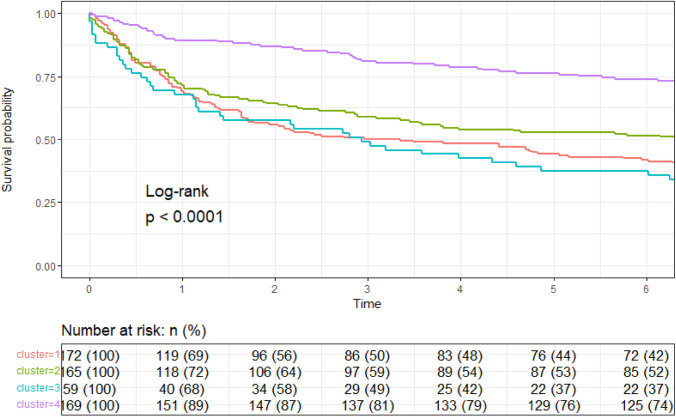
Clusters and risk of death or 6-month HF readmission. Kaplan Meier curves

Concerning comorbidities, we observed higher prevalence of stroke and dementia in Cluster 1; whereas patient in Cluster 3 had higher proportion of patients with chronic kidney disease, atrial fibrillation, coronary artery disease and malnourishment. Furthermore, clusters differed also in terms of HF subtype proportion, based on ejection fraction, with patients in Cluster 3 more commonly HFrEF compared to the other subgroups (*p* < 0.001). Patients in Clusters 1 and 3 showed the highest mortality (52.3% and 49.2%, respectively), followed by Cluster 2 (38.5%) and Cluster 1 (17.8%). As shown in Fig. 3, as compared with Cluster 4, by Cox multivariable regression analysis, Clusters 1 and 2 showed 3.5-higher risk of 6-month adverse outcome, whereas patients in Cluster 2 had a 2.6 (Table 3).

**Table 3:** Six-month mortality of HF readmission according to the k-means clusters

	Univariate			Multivariable^a^		
	H.R	95% CI	*p* value	H.R	95% CI	*p* value
Cluster 1	3.87	2.56–5.86	< 0.001	3.53	2.30–5.39	< 0.001
Cluster 2	2.73	1.77–4.22	< 0.001	2.59	1.66–4.07	< 0.001
Cluster 3	3.81	2.29–6.36	< 0.001	3.75	2.25–6.27	< 0.001

Cox multivariable regression analysis

^a^Age and sex adjusted. Reference: Cluster 4

### Hierarchical clustering (HC)

As secondary analysis we further run HC analysis to confirm the strength of our findings. Alike k-means clustering, by silhouette methods four and six clusters were found to be the most accurate numbers of clusters according to the dataset (Supplemental Fig. 2). By visual dendrogram analysis and given the small sample size, four cluster were chosen for the analysis. As shown in Supplemental Table 1, according to CFS, CCI, and CFS, patients in Cluster 1 were non-frail with low levels of BNP (i.e., Cluster 4 in k-means), Cluster 2 low BNP pre-frail to frail, pre-frail-to frail (k-means Cluster 2), Cluster 3 high BNP, pre-frail-to frail (k-means Cluster 3), Cluster 4 very frail patients with high BNP (k-means Cluster 1). We observed an higher 6-month mortality or re-hospitalization in Cluster 4 (77.6%) followed by Cluster 3 (67%), Cluster 2 (60.2%), and Cluster 1 (41.8%). As shown in Supplemental Table 2, by age- and sex-adjusted Cox regression analysis, as compared to Cluster 1, patients in Cluster 4 showed almost a threefold higher risk of the composite outcome (aHR 2.68, 95% CI 1.84–3.91).

## Discussion

In a group of older patients hospitalized for acute decompensated heart failure, frailty degree, burden of comorbidities and levels of BNP emerged as independent markers of 6-month adverse outcomes. By random forest analysis, age, BNP, frailty degree, comorbidity burden, and creatinine at discharge were the most important predictors of 6-month HF re-hospitalization or death following discharge for acute HF. We leveraged a machine-learning-based analysis strategy and two distinct clustering analyses, able to identify four distinct clinical phenogroups of acute decompensated older patients interventions to prevent adverse outcomes. Especially for the pre-frail patients, targeted interventions to improve the global functional status may improve their prognosis after discharge.

During the past 10 years, a variety of approaches have been assessed to identify the mortality risk of HF patients, mostly using readily available demographic, clinical, and laboratory data points [30]. The importance of stratifying the risk of future adverse outcomes, especially in older adults with HF, should be helpful to individualize the ones who might benefit more from aggressive treatment and closer follow-up. Many studies on predictive markers for outcomes in patients with HF have been published but most of the current calculators are inaccurate for older adults since they generally underestimate absolute mortality risk in frail patients [3, 30].

According to our data, BNP, CFS, and CCI were found to be significantly associated with increased 3-month mortality risk or re-hospitalization for HF decompensation.

By k-means clustering, we observed high rates of adverse outcomes in Clusters 1 and 3, indicating, respectively, those who were frail, comorbid, or pre-frail/comorbid with high levels of BNP. That class of individuals were more likely to have a HFmrEF or HFrEF, than the other clusters. Our data confirmed that BNP was a strong predictor of adverse outcomes in ADHF; also after adjustment for potential confounders. As known biomarkers maintain a major role in the prognostic assessment of HF patients. In particular, BNP, produced by the myocardium primarily in response to volume overload and increase in wall stress, and its inactive metabolite N-terminal pro-B-type natriuretic peptide (NT-proBNP), are established prognostic markers in patients with heart failure and reduced ejection fraction (HFrEF) [31]. Several studies showed that biomarkers like NT-proBNP could have a major role in the prognostic assessment of HF patients [9, 32], even in a geriatric population [33]. Accordingly, the highest mortality was found in patients with high proportion of HFrEF and high levels of BNP.

Interestingly, although individuals in Clusters 2 and 3 shared same mean age (85.5 years) and degree of frailty (median CFS = 6), the latter suffered from almost 10-percent lower mortality. These finding could be explained by the fact that the degree of chronic kidney disease (71.2% vs 41.4%), HFrEF (32.2% vs 14.8%), higher median BNP (4100 vs 593 pg/ml) and creatinine, thus possibly depicting patients with advanced HF with cardio-renal syndrome. On the other hand, patients in Cluster 4 showed high proportion of CCI, which is a well-recognized prognostic tool, they were the group of individuals with the lower rates of severe outcomes. This is not surprising as they were the fittest group, according to the CFS, therefore it is possible to speculate on their higher ability of recovering following an acute hospitalization. Frailty is a common geriatric syndrome, characterized by the decline of physiological systems and reserve with inadequate response to minimal environmental stressors, leading to higher clinical vulnerability. This syndrome is frequent in older patients with heart failure, and both frailty and heart failure share common mechanistic features, including strong relations with a high burden of comorbidities, inflammation, and sarcopenia [5]. The role of frailty has been increasingly recognized in cardiovascular diseases, and it has been recently identified as an independent factor for long‐term mortality and hospital readmission in nondependent older adults with heart failure [34, 35]. Therefore, the inclusion of frailty determinants into cardiac prognostic models has been progressively applied [36, 37]. Although there is consensus regarding the conceptual definition of frailty, there is no consensus on how frailty should be measured. Currently, there are several approaches to the assessment of frailty but many of these measures are not integrated into routine care for all patients since they are time‐consuming and of specialist expertise. In a recent study, Sze et al. evaluated commonly used frailty tools and they concluded that CFS might be the preferred method for a rapid evaluation of frailty in HF patients, as its prognostic value was comparable with that of complex assessment tools or physical tests [5].

The present study is not free from limitations: starting from the “single-center” investigation; therefore, further multicenter evidence is warranted to validate the prognostic significance of clustering based on CFS and BNP level older patients with ADHF. Moreover, caution must be taken due to the small/medium size of the dataset potentially affecting the evaluation of the feature importance, and the large confidence intervals of the determinants. Therefore, an external validation using larger cohorts of elderly with HF is warranted to enhance the generalizability of the results. Nonetheless, the clinical relevance of the endpoint predictors was further confirmed by random forest analysis and the hierarchical agglomerative clustering, thus strengthening our findings. In addition, given the retrospective nature of the analysis, it was not possible to propose corrections or adjustments regarding the intrinsic subjectivity of CFS from different clinicians. some essential features, characterizing the population and the pathology, such as body mass index, which may exert a significant impact on the short-term outcome, were not considered in the analyses. Moreover, no data are available on the etiology that led to hospitalization for decompensated HF and that may have affected the short-term composite endpoint. Similarly, it was not possible to collect data on chronic therapies with effects on HF mortality, nor on drugs that could induce changes in the values of the considered biomarkers. Data on the percentage of patients eventually referred to cardiovascular rehabilitation programs, also potentially affected by the global status [38], were not collected.

Nonetheless, this study analyzes a large cohort of older patients referred to the emergency departments, following them for a 6-month time interval, analyzing aspects often lacking in the scientific literature in the geriatric field, such as frailty and comorbidity load. In the present study, we confirm the importance of the integration of frailty assessment using CFS. Since CFS combines clinical judgment with objective measurement and can be easily conducted, it represents a practical way of screening frailty in routine assessment, especially in acute care setting [29]. The merit of proposing an innovative approach, computerized but easily applicable in almost all hospitals, is also worth mentioning, to generate clusters of patients on the basis of simple values, aimed at creating treatment paths adapted to the clinical condition and prognosis of each older individual, whose approach is often complicated by an enormous phenotypic heterogeneity that alters the global picture.

In conclusion, our study indicates that frailty, comorbidity burden, and BNP levels are independent markers of 6-month adverse outcomes in older patients with acute decompensated heart failure. The machine-learning-based clustering strategy allowed the identification of four distinct phenogroups of acute decompensated elderly patients that were characterized by differences in frailty, comorbidity burden, and short-term prognosis. Physicians should assess frailty, BNP levels, and comorbidity burden to identify high-risk patients who require closer monitoring and interventions to prevent adverse outcomes. For pre-frail patients, targeted interventions to improve the frailty global functional status of patients may improve their prognosis after discharge.

### Supplementary Information

Below is the link to the electronic supplementary material.

Supplementary file1 (DOCX 17 KB)

## Data Availability

The data that support the findings of this study are available from the corresponding author, upon reasonable request.
