# Supplementary material for: Predicting mortality and re-hospitalization for heart failure: a machine-learning and cluster analysis on frailty and comorbidity
Source: Aging Clin Exp Res. 2023 Oct 18;35(12):2919–28. doi: 10.1007/s40520-023-02566-w (PMC10721693; doi:10.1007/s40520-023-02566-w)
Supplement: Supplementary file 1 — Supplementary file1 (DOCX 17 KB) [file 40520_2023_2566_MOESM1_ESM.docx]

|  | All patients  N=571 | Cluster 1  N=141 | Cluster 2  N=302 | Cluster 3  N=61 | Cluster 4  N=67 |
| --- | --- | --- | --- | --- | --- |
| Female (%) | 313 (54.8) | 58 (41.1) | 193 (61.0) | 29 (47.5) | 33 (49.2) |
| Age mean, years (SD) | 86.3 (6.3) | 85.0(6.4) | 86.9(6.3) | 85.7(6.3) | 86.7(5.2) |
| CFS median (IQR) | 6 (4) | 3 (2) | 6 (1) | 4 (4) | 7 (1) |
| CCI median (IQR) | 5 (4) | 6 (2) | 4 (4) | 4 (3) | 8 (1) |
| Creatinine median (IQR) | 1.22 (0.85) | 1.32 (0.84) | 1.02 (0.70) | 1.45 (0.8) | 1.32 (1.33) |
| BNP median, pg/ml (IQR) | 650 (931) | 475 (723) | 593 (743) | 3736 (1585) | 798 (698) |
| Composite endpoint (%) | 334 (58.4) | 59 (41.8%) | 182 (60.2) | 41 (67.2) | 52 (77.6) |

**Supplemental Table 1: Characteristics of Hierarchical Clustering subgroups.**

**Supplemental Table 2: Hierarchical Clustering Cox Regression Analysis**

|  | Univariate | | | Multivariable* | | |
| --- | --- | --- | --- | --- | --- | --- |
|  | **H.R.** | **95%CI** | **P-value** | **H.R.** | **95%CI** | **P-value** |
| Cluster 2 | 1.84 | 1.37 – 2.46 | <0.001 | 1.81 | 1.34 – 2.44 | <0.001 |
| Cluster 3 | 2.16 | 1.45 – 3.22 | <0.001 | 2.18 | 1.46 – 3.26 | <0.001 |
| Cluster 4 | 2.89 | 1.98 – 4.20 | <0.001 | 2.68 | 1.84 – 3.91 | <0.001 |

*age and sex- adjusted.

Ref-Cluster 1
